# Supplementary material for: A novel mouse model expressing human forms for complement receptors CR1 and CR2
Source: BMC Genet. 2020 Sep 9;21:101. doi: 10.1186/s12863-020-00893-9 (PMC7487969; doi:10.1186/s12863-020-00893-9)
Supplement: Supplementary file 1 — Additional file 1. [file 12863_2020_893_MOESM1_ESM.docx]

**Supplemental Figures**

**
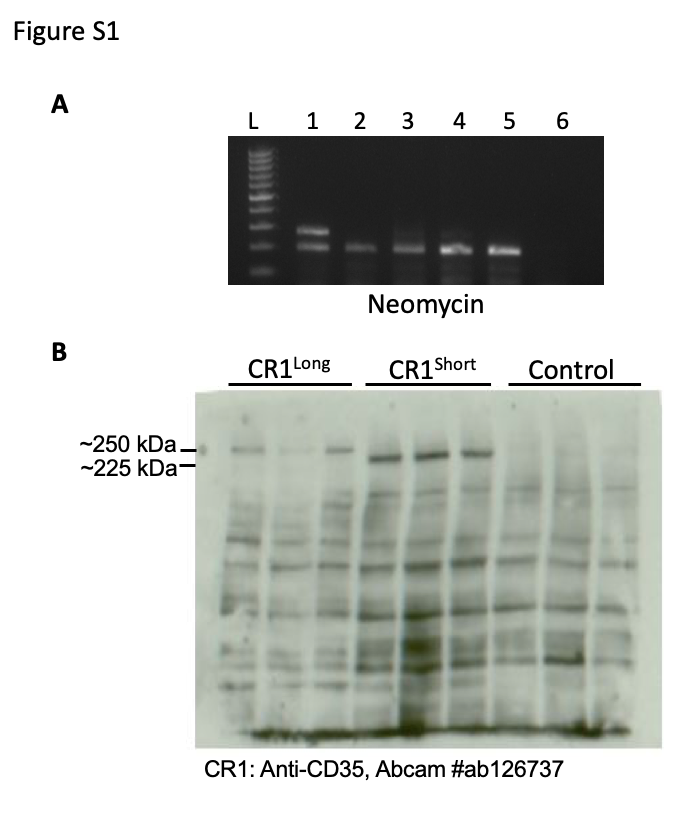
**

**Figure S1:** (**A**) Presence of Neomycin cassette in heterozygous B6.*CR2/CR1^long/+^* with Neomycin cassette but not mice after excision with *Phi*. 1 – heterozygous. B6.*CR2/CR1^long/+^* with Neomycin cassette. 2 – homozygous B6.*CR2/CR1^long/long^*. 3 – homozygous B6.*CR2/CR1^short/short^*. 4 – homozygous B6.*CR2/CR1^KO/KO^*. 5 – B6. 6 – Water. (**B**) Full western blot depicted in **Fig. 4F**.


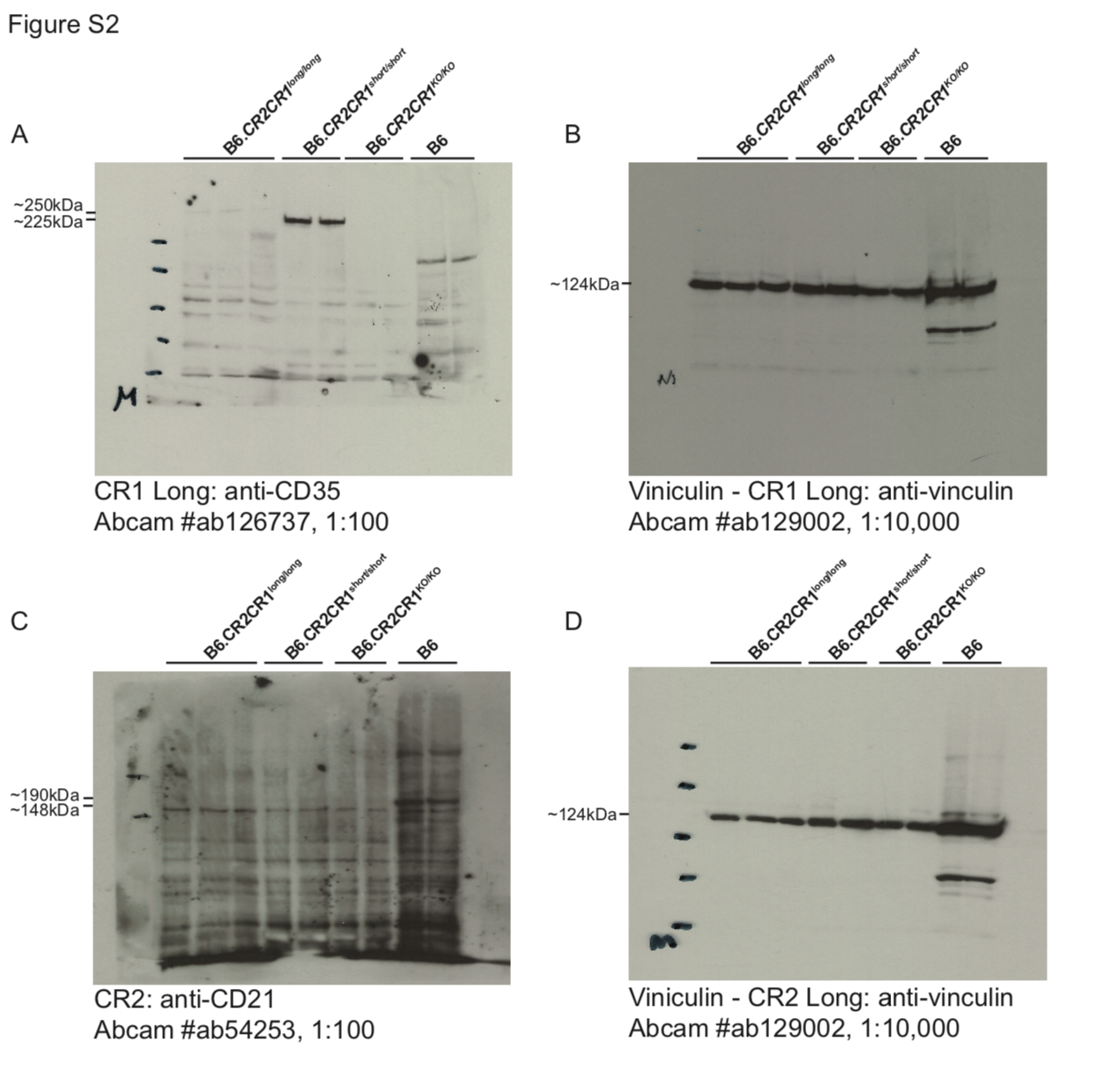


**Figure S2:** (**A-B**) Full western blots depicted in **Fig. 5A.** (**C-D**) Full western blots depicted in **Fig. 5D**.

**Supplemental Files and Tables (provided separately)**

Supplemental file CR1 and CR2 protein alignments

Table S1 DE genes Spleen_CR2CR1long_vs_B6.xlsx

Table S2 DE genes Spleen_CR2CR1short_vs_B6.xlsx

Table S3 DE genes Spleen_CR2CR1KO_vs_B6.xlsx

Table S4 DE genes Brain_CR2CR1long_vs_B6.xlsx

Table S5 DE genes Brain_CR2CR1short_vs_B6.xlsx

Table S6 DE genes Brain_CR2CR1KO_vs_B6.xlsx

Table S7 GO terms.xlsx
