# Supplementary figures and images for: A novel mouse model expressing human forms for complement receptors CR1 and CR2
Source: BMC Genet. 2020 Sep 9;21:101. doi: 10.1186/s12863-020-00893-9 (PMC7487969; doi:10.1186/s12863-020-00893-9)

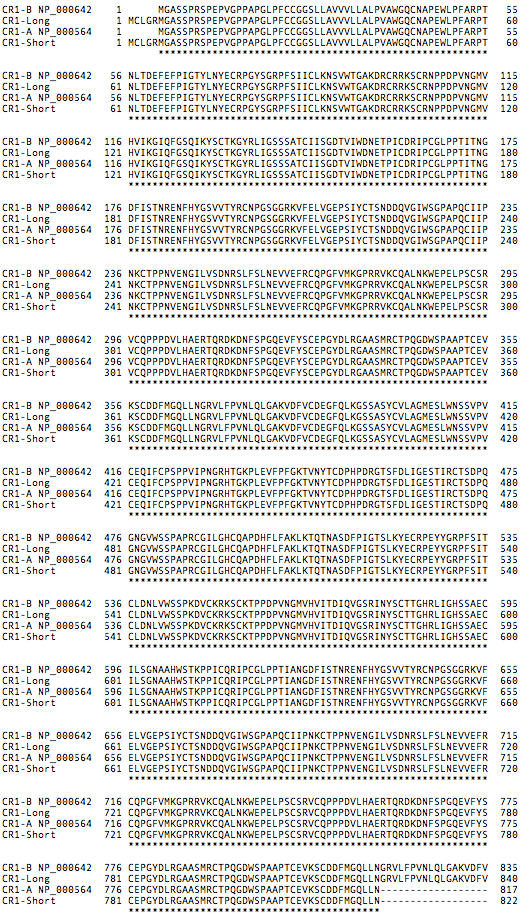

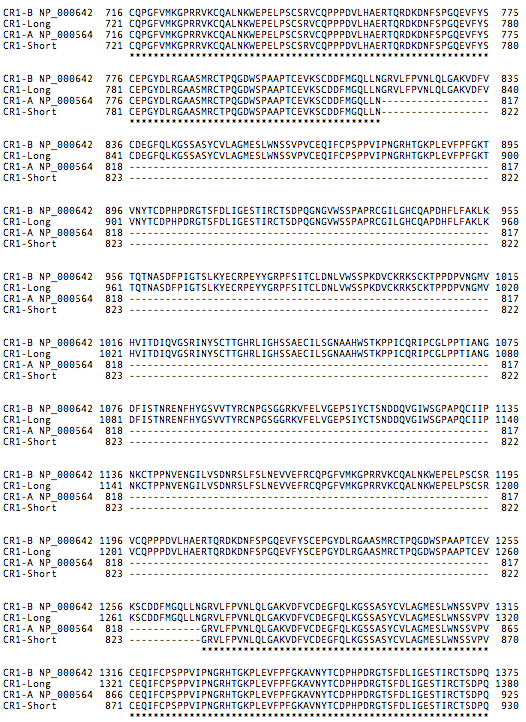

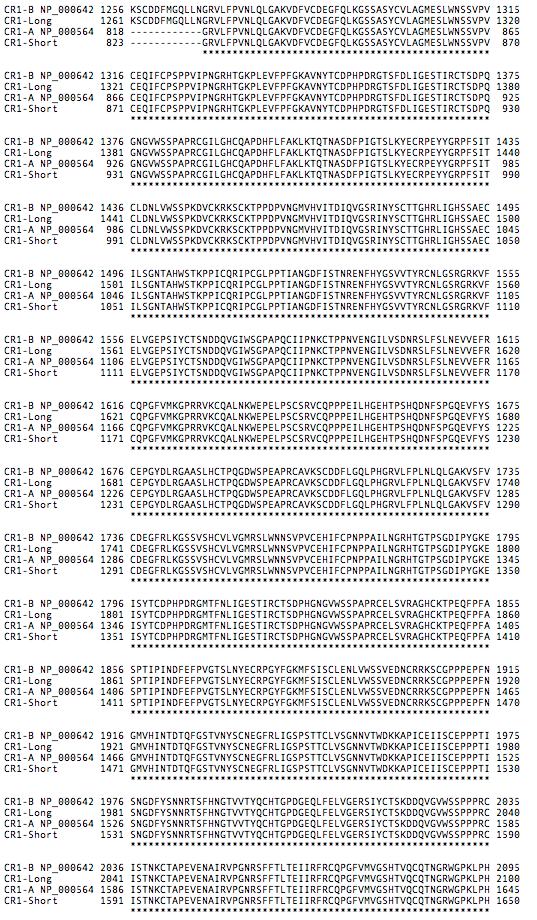


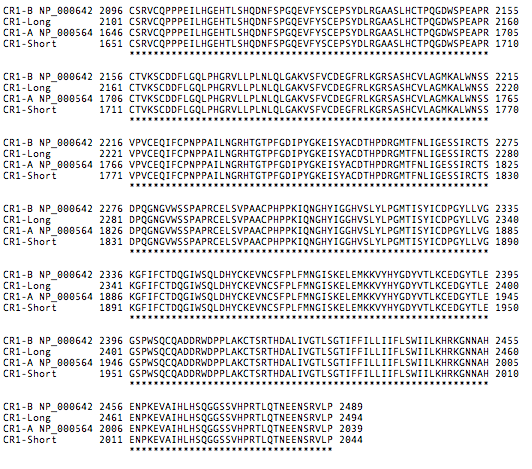


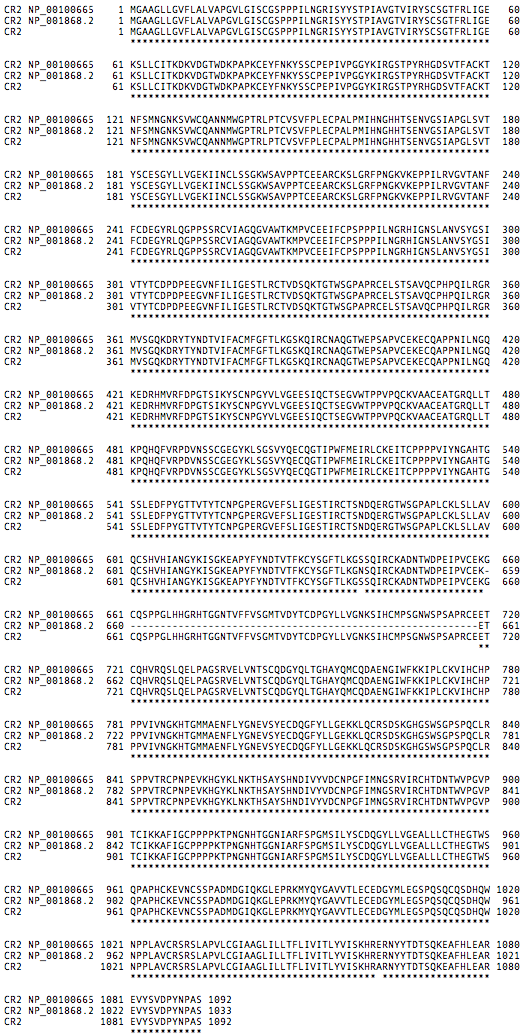

Supplement: Supplementary file 2 — Additional file 2. [file 12863_2020_893_MOESM2_ESM.docx]
